# Toward a New Primary Standardization of Radionuclide Massic Activity Using Microcalorimetry and Quantitative Milligram-Scale Samples

**DOI:** 10.6028/jres.126.048

**Published:** 2022-02-24

**Authors:** Ryan P. Fitzgerald, Bradley K. Alpert, Daniel T. Becker, Denis E. Bergeron, Richard M. Essex, Kelsey Morgan, Svetlana Nour, Galen O’Neil, Dan R. Schmidt, Gordon A. Shaw, Daniel Swetz, R. Michael Verkouteren, Daikang Yan

**Affiliations:** 1 National Institute of Standards and Technology, Gaithersburg, MD 20899, USA; 2 National Institute of Standards and Technology, Boulder, CO 80305, USA; 3 University of Colorado Boulder Boulder, CO 80309, USA

**Keywords:** alpha, beta, cryogenic detectors, mass metrology, microcalorimeter, radioactivity, radionuclide metrology, transition edge sensor

## Abstract

We present a new paradigm for the primary standardization of radionuclide activity per mass of solution (Bq/g). Two key enabling capabilities are 4π decay-energy spectrometry using chip-scale sub-Kelvin microcalorimeters and direct realization of mass by gravimetric inkjet dispensing using an electrostatic force balance. In contrast to traditional traceability, which typically relies on chemical separation of single-radionuclide samples, 4π integral counting, and additional spectrometry methods to verify purity, the system described here has both 4π counting efficiency and spectroscopic resolution sufficient to identify multiple radionuclides in the same sample at once. This enables primary standardization of activity concentrations of mixed-radionuclide samples. A major benefit of this capability, beyond metrology, is in assay of environmental and forensics samples, for which the quantification of multiplenuclide samples can be achieved where presently inhibited by interferences. This can be achieved without the need for chemical separations or efficiency tracers, thereby vastly reducing time, radioactive waste, and resulting measurement uncertainty.

## 1 Introduction

Measurements of activity (disintegration rate) of radionuclides are essential in revealing key information such as the age and origin of geologic or man-made materials, efficacy and safety of medical treatments, accountability of nuclear materials, and adequacy of cleanup efforts. To be of most value, such measurements often must be traceable to the becquerel (Bq, equivalent to s^−1^), the SI-derived unit that represents the decay rate for a given radionuclide. In practice, the massic activity of a solution must be realized in becquerel per gram (Bq g^−1^), that is, activity of the radionuclide per total mass of solution, such that subsequent sampling by mass (or volume, assuming density is known) will have known activities. The National Institute of Standards and Technology (NIST) and other national metrology institutes are foundational to such traceability through the realization of primary standards for the becquerel [1, 2] (and kilogram) and dissemination through means such as reference materials, calibrations, measurement assurance programs, and proficiency testing programs. The roles of these programs are particularly important for demonstrating measurement capabilities where potential bias and uncertainty come not only from primary standards, but also from radionuclidic interferences and issues involving addition of chemical tracers and enriched isotopic tracers [3]. Similar considerations can be found in mass spectrometry, which is susceptible to isobaric interferences (*e.g.*, ^241^Am and ^241^Pu), and where reference materials certified for activity are used as isotopic tracers by invoking half-lives to calculate the amount of a nuclide.

One challenge with the traditional primary standardization methods is that they typically are limited to (nearly) pure single-radionuclide solutions and rely on multimethod analysis [1]. Most primary methods, such as 4π α/β-γ coincidence counting, measure only total activity and thus require one or more additional spectroscopic methods (*e.g.*, γ-ray spectrometery) to make corrections for any radionuclide impurities [4]. Likewise, mass spectrometry can be used to determine the relative proportions of isotopes and, using isotope dilution, the number of radionuclide atoms in a sample, but this technique is sensitive to isobaric interferences, which can necessitate secondary methods (such as α spectrometery) for making impurity corrections [5, 6].

The paradigm envisioned in this paper is to develop a capability to (1) replace the existing multistep method (absolute counting + spectroscopic purity analysis) with a single measurement that achieves both results with lower uncertainty and (2) provide the same capability for mixed-nuclide samples that eliminates reliance on chemical separation, yield tracers, and enriched isotopic tracers (*e.g.*, those used to calibrate detection efficiency). The new paradigm will enable faster measurements with lower uncertainties, and it will work for some materials for which the current methods do not (due, *e.g.*, to a lack of isotopic tracers). The goal is to provide reference materials that do not yet exist and to reduce the uncertainty in activity for existing materials from order 1% down to order 0.1%. Additionally, this approach has the potential to measure systems for which isotopic tracers do not currently exist, *e.g.*, for ^226^Ra [7, 8], to standardize emerging α-decay-chain therapeutics [9], and to investigate error-causing radionuclides in materials used in microelectronics and quantum computing [10].

We propose to implement this technique, and thereby realize massic activity (Bq g^−1^) in a fundamental way, by absolute decay-energy spectrometery (ADES) using cryogenic transition edge sensor (TES) microcalorimeters, applied to quantitatively prepared radioactive sources. Decay-energy spectrometery (DES) is calorimetry, but it is a radical departure from classical radionuclide calorimetry, including “cryogenic microcalorimetry” (which was microwatt calorimetry at 8 K) [11]. Whereas classical calorimetry measures the average power from a “hot” source (typically several gigabecquerels of activity), the radioactive decay products from each single decay event in DES are thermalized in a small absorber, and the decay energy (~1 pJ) from each event is measured with spectral resolving power of order >1000. Aside from DES, many TES measurements have focused on spectroscopy (*e.g.*, X-ray spectral energies and shapes) rather than spectrometry (intensities). In DES, both spectroscopy and spectrometry have been developed and applied in multiple fields, including nuclear safeguards, forensics, medicine, and nuclear decay data. A recent review of the topic [12] cited the first DES measurement in 1992 [13] and the first TES-based DES measurement in 2000 [14], both of which aimed to put a limit on neutrino mass by measuring the end-point energy of beta decay spectra. An important milestone in spectrometery was the first measurement of the isotopic ratio of plutonium isotopes [15, 16], which agreed with traditional mass spectrometery. Realizing massic activity based on ADES will require numerous innovations in design and analysis to achieve sufficient accuracy. These innovations include performing the following tasks in an SI-traceable fashion: (1) gravimetrically linking sample mass to starting solutions, (2) linking observed microcalorimeter count rate to total decay rate, and (3) quantifying specific isotopes from potentially complex spectroscopic signatures.

## 2 Experiment

**Fig. 1:**
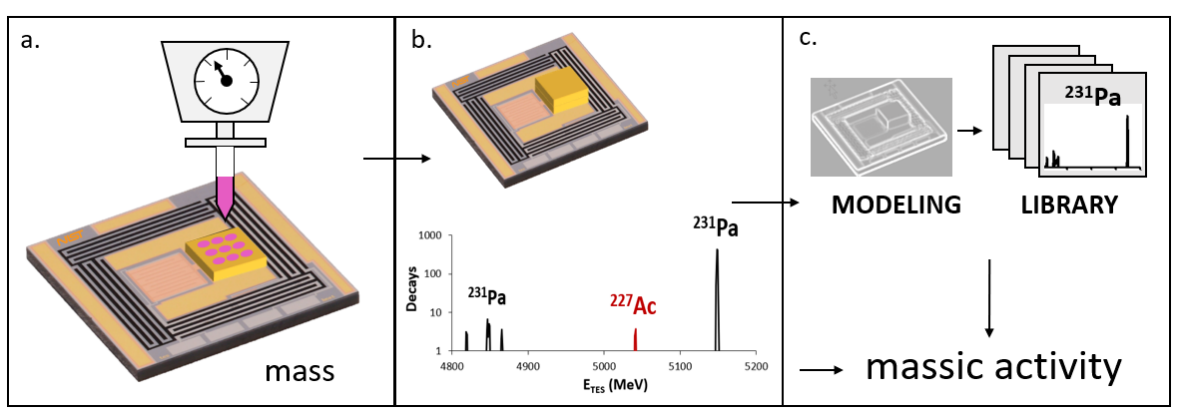
Measurement process. (a) Mass of radionuclide solution (pink) deposited on substrate (gold) is determined by gravimetric inkjet dispenser. (b) Top layer of substrate is added for 4π decay-energy spectrum (mixed ^231^Pa/^227^Ac source simulated). (c) Nuclide is identified and quantified using library of measured and modeled radionuclide decay signatures, combined with mass to output massic activity.

The illustration of the ADES measurement process shown in Fig. 1 includes quantitative source preparation, 4π DES measurements, and automated spectral analysis using extensive modeling.

Two key developments enable ADES. The first key development is the advancement of cryogenic microcalorimeters with ultralow noise floors and excellent energy resolution. Importantly, these sensors can be fabricated to have enough recovery time to resolve consecutive radioactive decays at high enough count rates to achieve precise counting measurements. Microcalorimeters are thermal sensors that rely on sub-Kelvin temperatures to achieve electronvolt-scale resolution. Because they are thermal sensors, a wide variety of energy sources can be efficiently coupled to microcalorimeters; DES relies on embedding radioisotopes in a substrate that is thermally coupled to a microcalorimeter to achieve 4π geometric efficiency. NIST develops and deploys spectrometers based on TES microcalorimeters amplified by a series of superconducting quantum interference devices (SQUIDs). Historically, the focus has been on developing TES for external-source applications spanning near-infrared [17] to γ-ray [18] parts of the electromagnetic spectrum, and notably including x-ray wavelengths [19]. Non-absolute TES-based DES was developed by a collaboration between NIST and the Los Alamos National Laboratory (LANL) [15]. DES has been developed with TESs and other ultralow-temperature sensors such as metallic magnetic calorimeters (MMCs) [20] for a variety of applications such nuclear safeguards, forensics, and nuclear decay data [12, 21]. For practical ADES, TESs must be able to handle larger heat-capacity absorbers and to absorb decay energy from energetic particles while also achieving fast thermal recovery. Present DES count rates are limited to ~1 s^−1^. However, for metrology, the goal is to acquire enough counts to achieve 0.05% statistical uncertainty in the average count rate. For a Poisson process, this requires 4 × 10^6^ decays, which corresponds to an average decay rate of about 6.6 s^−1^ for a counting period of 1 week or 139 s^−1^ for a counting period of 8 h. In many cases, longer count periods are acceptable, and multiple sources can be measured at once to reduce count rate needed per sample. However, for the highest count rate goal considered, to keep pileup and dead-time effects manageable, the TES thermal recovery time constant after a pulse should be significantly shorter than the inverse of the count rate, which is 7.2 ms. (For reference, the typical time constant for a TES-based alpha DES measurement is approximately 30 ms [15].) The ability to measure higher-activity sources will be important for measuring medical radionuclides, which typically have half-lives of a few hours, and also for characterizing radioactive impurities or isotopic ratios, in which cases one may need to make precise measurements of a lower-activity radionuclide in the presence of a high count rate from a higher-activity radionuclide [22, 23]. Depending on the application, long count times, faster detectors, multiplexing (measuring multiple samples at once), or a combination thereof, will be required to achieve the necessary precision.

The second key development is the 2019 redefinition of the kilogram in the SI system of units [24], which opened new paths to high-accuracy realization of milligram-scale masses. A DES source must be very small (typically micrometer-thickness Au foils and areas < 10 mm^2^) because larger sources reduce the temperature rise due to a decay and therefore limit energy resolution. This in turn constrains the size of the sample of radioactive solution to be ≈ 1 μL, corresponding to about 1 mg of mass. Measuring the mass of such a small, radioactive, aqueous sample presents multiple challenges (see Sec. 3), including calibration of an appropriate balance. We plan to use a combination of advanced inkjet printing [25] and a method of directly realizing milligram-scale masses with electrical metrology [26] to enable ADES.

A goal of this work is to achieve and validate 100% counting efficiency for α-emitting radionuclides. If ADES is performed such that 100% of the energy is captured for each radioactive decay, then the events from all decay branches will occur in a single narrow peak at the Q value (where Q is the difference in rest mass between the parent and offspring atoms). In this case, the energy resolution of a microcalorimeter would be sufficient to enable perfect isotopic identification by a simple region of interest (ROI)–based spectral analysis. While alpha particles can be stopped with 100% efficiency, beta particles and gamma radiation can escape the necessarily small DES sources. Additionally, the bulk material (often Au) of the DES source can be excited to emit X-rays, which may also escape the source. Nuclear decay chains introduce correlated events and therefore do not allow a simple Poisson-based correction for detector dead time. Therefore, for ADES to be applied to the widest variety of mixed radionuclides, we plan to use comprehensive source and detector modeling to identify radionuclides and extract corresponding activities. In many cases, an automated analysis is envisioned.

### 2.1 Detectors

Within the detector system, the microcalorimeter, the 4π absorber, the thermal connection between the two, and the downstream SQUID amplification need to be optimized for ADES. The TES is voltage-biased in an electric circuit. When decay energy in the form of kinetic energy of particles or photon energy is dissipated in the absorber that is in thermal contact with the TES, the TES resistance increases sharply due to heating. This causes a *pulse* of reduced current signal, the amplitude of which is measured to calculate the input energy. Within resolution constraints, the microcalorimeters will be optimized for speed, both in the rising edge and falling tail of the heat pulse.

**Fig. 2:**
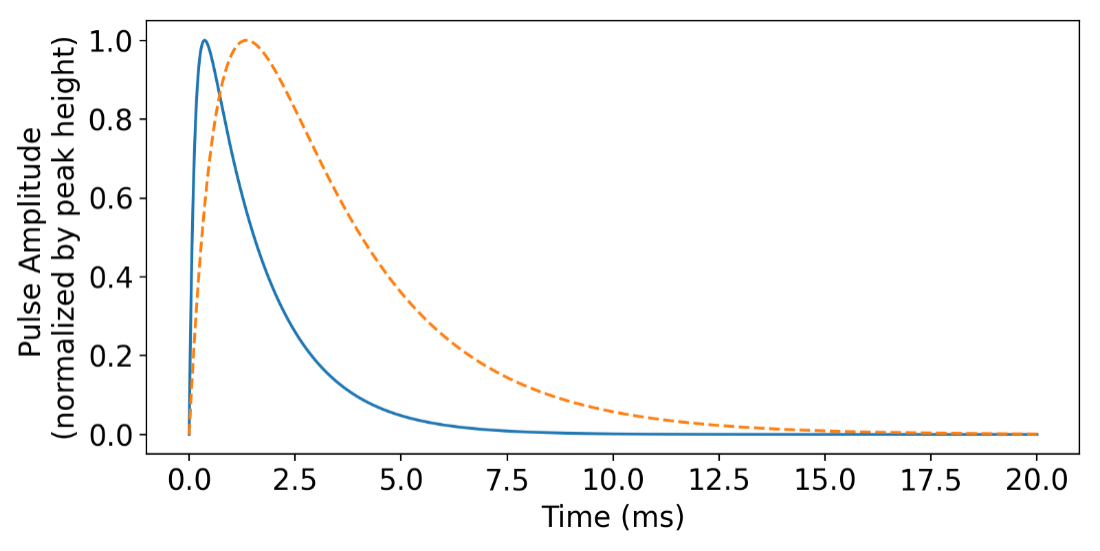
Simulated current signal, shown as positive by convention and normalized such that the peak height is 1, for slower (dashed orange line) and faster (solid blue line) TES sensors. The slower TES has 5 times more inductance and 2.5 times higher thermal conductivity than the faster TES.

The pulse shape is important for extracting high-resolution energy spectra and quantifying the fraction of pulses missed or discarded (dead-time) during analysis [27]. The TES pulse shape may be modeled with a set of electrothermal differential equations [28]. Figure 2 shows two example pulses. The thermal conductivity to the bath and the inductance in series with the TES are varied to control the rise and fall times of the signal. Notably, TES detectors in general have a much slower response (on the order of milliseconds) compared to the detectors more commonly used for primary standardization (<1 µs). The combination of high count rate and large absorbers desired for ADES will require TES with much higher thermal conductivity than is typical for other applications.

Detector modeling is being extended to include multibody effects of the TES, the 4π source absorber, and the links between them. Some designs being studied both for TES and MMC allow the absorber to be on a separate chip, connected by gold wires, with an advantage of allowing the source to be changed without damage to the sensor chip [16, 21].

### 2.2 Source Preparation

Two major avenues of source preparation research are on efficient coupling of radioactive decay energy to the microcalorimeter sensor and quantification of dispensed mass. For the first avenue, one significant concern is the creation of large salt crystals when drying an aqueous deposit of dissolved radionuclides, as such a condition leads to degraded energy resolution [15]. For dilute solutions, inkjet patterning may be helpful in controlling the size of the resulting dried deposit. Mechanical kneading of foils has proven to be effective at breaking up crystals to achieve high resolution, but so far this has led to unquantified radionuclide sample loss, so it may not be appropriate for ADES. Nanopatterned substrates, such as nanoporous gold, are also being explored to constrain crystal size [29]. The distribution of the sample within an absorber material can lead to more or less energy loss due to escape radiation and has significant implications for isotopic identification. This can be controlled by over encapsulating the source with additional material. Adequate quantitative source preparation will likely involve a combination of these methods as well as checking for losses on tools and surfaces. A further approach is to operate in a mixed mode in which high-resolution nonquantitative and lower-resolution quantitative sources are prepared from the same material and measured with separate sensors.

**Fig. 3:**
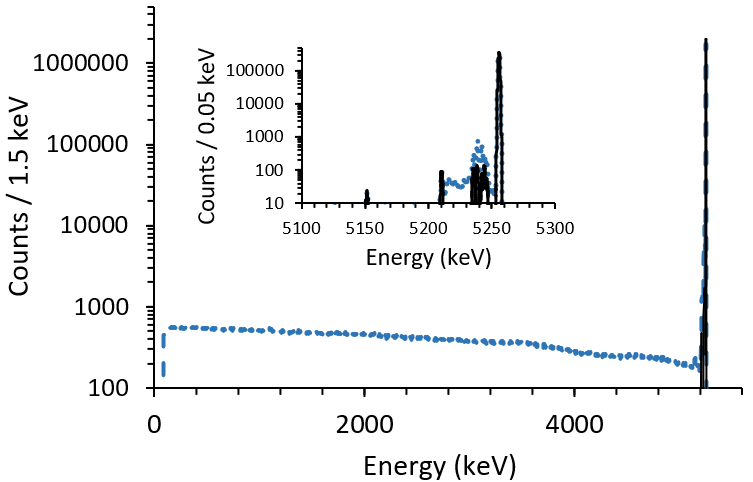
Monte Carlo simulations of DES spectra for Pu-240 in two geometries: source radionuclides either distributed uniformly in absorber (dashed blue) or else centered in 30 μm thick gold foil absorber (solid black), with peak-area counting efficiency of about 85.7% and 99.8%, respectively. For the uniform source, the spectrum is continuous down to 87 keV (energy of the U-236 recoil nucleus). Inset shows detail of peak region, including photon escape events, which are more numerous for the uniform source.

Radionuclide decay and radiation transport modeling, using Geant4 Monte Carlo simulation libraries, are also being carried out to optimize absorber design and determine counting efficiencies [27]. The Monte Carlo simulations include radioactive decay, nuclear and atomic cascades, radiation transport, and interactions. Future work may include phonon creation and transport. A particular focus is on choosing the material and geometry needed to stop particles (α, e^−^) and absorb or let pass higher-energy photons. Figure 3 shows simulated spectra for an ADES source consisting of Pu-240 embedded in a 30 μm thick Au foil. The radionuclide is either distributed evenly through the foil, approximating the results of the sample kneading method used to achieve the highest resolution DES results so far [30], or deposited as a point in the center of the foil. In the evenly distributed case, the primary peak contains only 85.7% of the counts, primarily due to beta particles depositing only a fraction of their energy in the foil. In the central point case, 99.8% of the counts appear in the primary peak, and the remaining approximately 0.2% appear in escape peaks with well-known energies ~100 keV below the primary peak. In multi-isotope samples, counts outside of the primary peak of one isotope could interfere with the main peak of a second isotope. These results indicate that for low uncertainty in the activity, ADES will require some combination of a central positioning of the radioisotopes and, in most cases, a more complex spectral analysis.

The second major effort on source preparation is focused on quantification of the mass of dispensed solution. The well-established method for radionuclide source preparation is to determine the dispensed mass by difference using pycnometers (~5 mL disposable polyethylene bottles with drawn-out necks to form capillaries) [31]. The method of determining the mass of dispensed solution by weighing the pycnometer before and after dispensing can achieve uncertainties down to 0.05% but only for masses >20 mg. An alternative metrological approach to determining dispensed solution at the needed 1 mg level is by inkjet, calibrated by weighing the dispensed drop itself on an ultramicrobalance, and limited to 1% uncertainty by evaporation of that drop and inkjet dispenser calibration repeatability [25]. Our main approach is to combine the two methods by using an inkjet with built-in balance to measure dispensed mass by difference (not weighing the drop itself), as if the pycnometer were attached to the balance. The viability of this approach is greatly increased by the ability within the SI to realize the kilogram using electrical standards traceable to Planck’s constant using quantum standards. The proposed method includes an electrostatic force balance (EFB) in-line with the inkjet dispenser. The EFB directly realizes mass *in situ* using electrical metrology (producing a primary realization of force, which, combined with gravity [*g*], gives mass) [26], with relative uncertainties an order of magnitude lower than calibrations from other masses. In weighing the dispenser by difference, instead of weighing the drop of liquid, we drastically reduce the correction for evaporation. Preliminary results have established the accuracy of a milligram dispensed droplet mass measurement by direct comparison to a reference mass and indicate this uncertainty can be constrained to <0.03% [32]. The embedded mass reference effectively eliminates the repeatability uncertainty. Our estimated uncertainty budget for determining the mass is shown in Table 1. Validation can be performed by isotope dilution mass spectrometery (IDMS) based on systems for which high-purity isotopic samples are available (such as gadolinium) or by liquid scintillation (LS) on gravimetrically linked solutions.

**Table 1:** Estimated standard uncertainty (*u*) for dispensed aqueous mass of 1 mg using existing (current) and dispensing-balance method (proposed).

**Source**	**Current *u***	**Proposed *u***
Calibration	0.1%	0.002%
Evaporation	0.2%	<0.03%
Repeatability	0.3%	<0.04%
Combined	0.4%	<0.05%

### 2.3 Analysis

Analysis refers here primarily to the process of taking detector output signals and extracting the activity of various radionuclides in the source, which, when combined with the mass of solution deposited in the source, returns the massic activity of the starting solution. Two main aspects of analysis are pulse processing and spectral analysis.

**Fig. 4:**
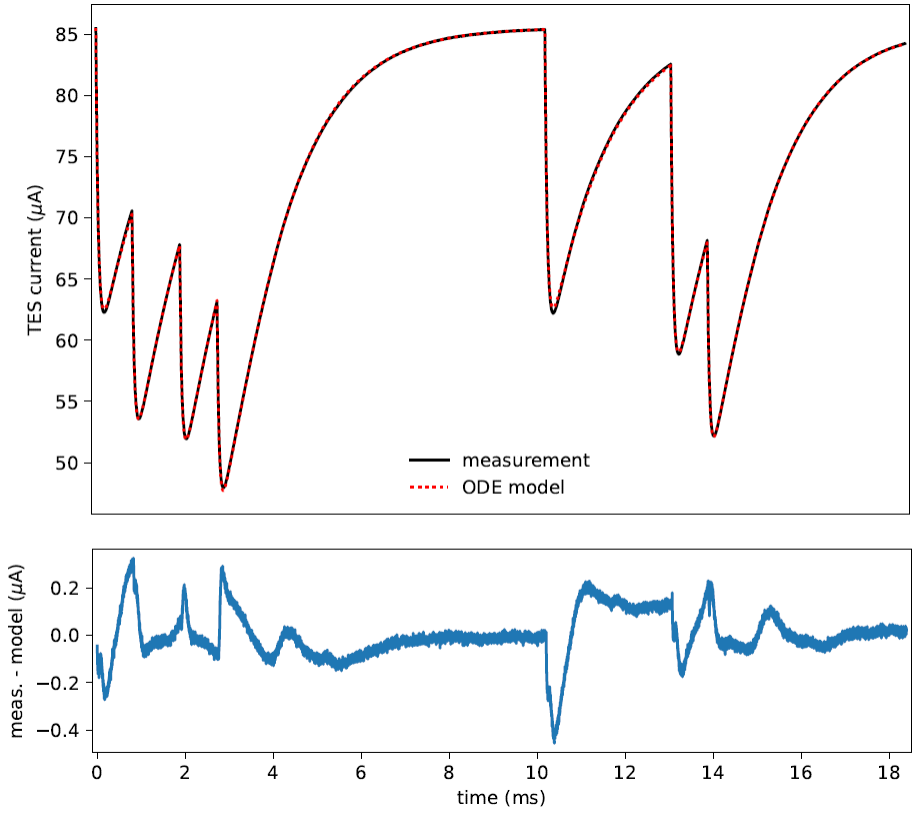
Example of seven piled-up pulses from Mn X-ray fluorescence lines showing measured TES current and an ordinary differential equations (ODE) model (top) and the difference between the measurement and the model (bottom), with a root-mean-square value of 1.4 × 10^−3^ in relative current.

Pulse processing refers to converting pulses measured in current units to the decay energy. There are well-established methods for pulse processing to TESs, but these methods have an effective detector recovery time on the order of the pulse decay time [33, 34]. We are investigating new pulse processing methods with the goal of reducing the effective dead time to be comparable to the pulse rise time, which will allow the use of higher activity samples while maintaining absolute activity accuracy, with a likely trade-off in energy resolution. The problem is complicated for TESs compared to MMCs due to nonlinearities in the energy response. One approach is to obtain a highly accurate set of physical parameters with which to solve the TES differential equations. Then, one can fit a timestream for the energy and arrival time of each energy deposition. Figure 4 shows such a model fit to experimental data with multiple pulse-pileup features, where the physical parameters were determined using machine learning techniques. Ongoing research includes improving triggering algorithms to ensure that all pulses are accounted for and expanding the classes of physical models considered to decrease the residuals in the fit, and thereby improve the energy resolution.

Spectral analysis is conducted to identify nuclides and extract activities from the DES spectra (counts *vs*. absorbed energy). To achieve this, we are building a library of measured and simulated DES spectra (*e.g.*, Fig. 3) for a variety of source and detector geometries. The simulated spectra will be used to fit the measured DES spectra, such that the output is the activity of the radionuclide or radionuclides present in the spectra. This capability will be particularly important for medical nuclides, for which there is concern about the possible presence of impurities (*e.g.*, Ac-227 in Ac-225) [35], and as a complementary method to mass spectrometry, where isobaric interferences pose a challenge (*e.g.*, Pu-238/U-238) [22].

Validation of the analysis will be provided using single-nuclide reference materials that have been mixed gravimetrically to create sources with small impurities, high count rates, and other known challenging factors. We also envision the use of an on-chip heater to allow an end-to-end test of both analysis steps. We can simulate a decay chain, noting the time and thermalize energy associated with each decay, create corresponding thermal pulses with the heater, run the resulting data through the analysis chain, and compare the resulting activity to the known inputs of the simulation.

## 3 Outlook

The work described here is focused on creating a practical primary realization of the radionuclide activity per mass of solution (Bq g^−1^) for real-world samples. The likeliness of success is bolstered by a growing cohort of ultralow-temperature calorimetry efforts around the world, including those assessing isotopic ratios [15], fundamental physics [29], and the metrology of beta decay [36]. Our vision of complete assay of massic activity of radionuclides without the need for chemical tracers is a radical departure from the current state-of-the art, both for decay-counting methods and mass spectrometery. In addition, this method could be combined with mass spectrometry or other methods of realizing the mole to determine precise half-lives for radionuclides important to the nuclear industry, nuclear safeguards and forensics, and the earth sciences.
